# Geny: a genotyping tool for allelic decomposition of killer cell immunoglobulin-like receptor genes

**DOI:** 10.3389/fimmu.2024.1494995

**Published:** 2024-12-23

**Authors:** Qinghui Zhou, Mazyar Ghezelji, Ananth Hari, Michael K. B. Ford, Connor Holley, S. Cenk Sahinalp, Ibrahim Numanagić

**Affiliations:** ^1^ Department of Computer Science, University of Victoria, Victoria, BC, Canada; ^2^ Department of Electrical Engineering, University of Maryland, College Park, MD, United States; ^3^ National Cancer Institute, NIH, Bethesda, MD, United States

**Keywords:** KIR, bioinformatics, software, genotyping, computational biology, combinatorial optimization

## Abstract

**Introduction:**

Accurate genotyping of Killer cell Immunoglobulin-like Receptor (KIR) genes plays a pivotal role in enhancing our understanding of innate immune responses, disease correlations, and the advancement of personalized medicine. However, due to the high variability of the KIR region and high level of sequence similarity among different KIR genes, the generic genotyping workflows are unable to accurately infer copy numbers and complete genotypes of individual KIR genes from next-generation sequencing data. Thus, specialized genotyping tools are needed to genotype this complex region.

**Methods:**

Here, we introduce Geny, a new computational tool for precise genotyping of KIR genes. Geny utilizes available KIR allele databases and proposes a novel combination of expectation-maximization filtering schemes and integer linear programming-based combinatorial optimization models to resolve ambiguous reads, provide accurate copy number estimation, and estimate the correct allele of each copy of genes within the KIR region.

**Results & Discussion:**

We evaluated Geny on a large set of simulated short-read datasets covering the known validated KIR region assemblies and a set of Illumina short-read samples sequenced from 40 validated samples from the Human Pangenome Reference Consortium collection and showed that it outperforms the existing state-of-the-art KIR genotyping tools in terms of accuracy, precision, and recall. We envision Geny becoming a valuable resource for understanding immune system response and consequently advancing the field of patient-centric medicine.

## 1 Introduction

The natural killer (NK) cells are a critical component of the human innate immune system, which is the first line of host defense mechanisms against infections, viruses, and diseases. These cells are responsible for rapid response to various pathological challenges, such as viral-infected cells and cancerous cells (1–3). The NK cells are regulated by cell surface receptors that interact with major histocompatibility complex class I (MHC-I) molecules found on the surface of various cells in the body (4). These receptors are, in turn, encoded by Killer cell Immunoglobulin-like Receptor (KIR) genes, located on the human chromosome 19 within a 150kb region of the Leukocyte Receptor Complex (LRC), whose expression and interactions are essential for distinguishing between healthy and abnormal cells.

The KIR genes contribute to the wide array of immune responses observed among individuals due to their vast genetic diversity which also influences disease susceptibility (5). For that reason, KIR genes belong to the family of *highly polymorphic genes* and consequently harbor a myriad of known gene phases (also known as *alleles*, or in some cases *genotypes*) that are present among the human population (6). Importantly, this variation is not limited only to the coding regions; it also encompasses the regulatory regions that direct the expression of KIR genes. It has been proposed that this vast genetic diversity likely stems from the evolutionary pressures posed by constantly evolving viruses (7). Such intricate genetic architecture means that fewer than 2% of unrelated individuals share an identical KIR genotype (8).

**Figure 1:**
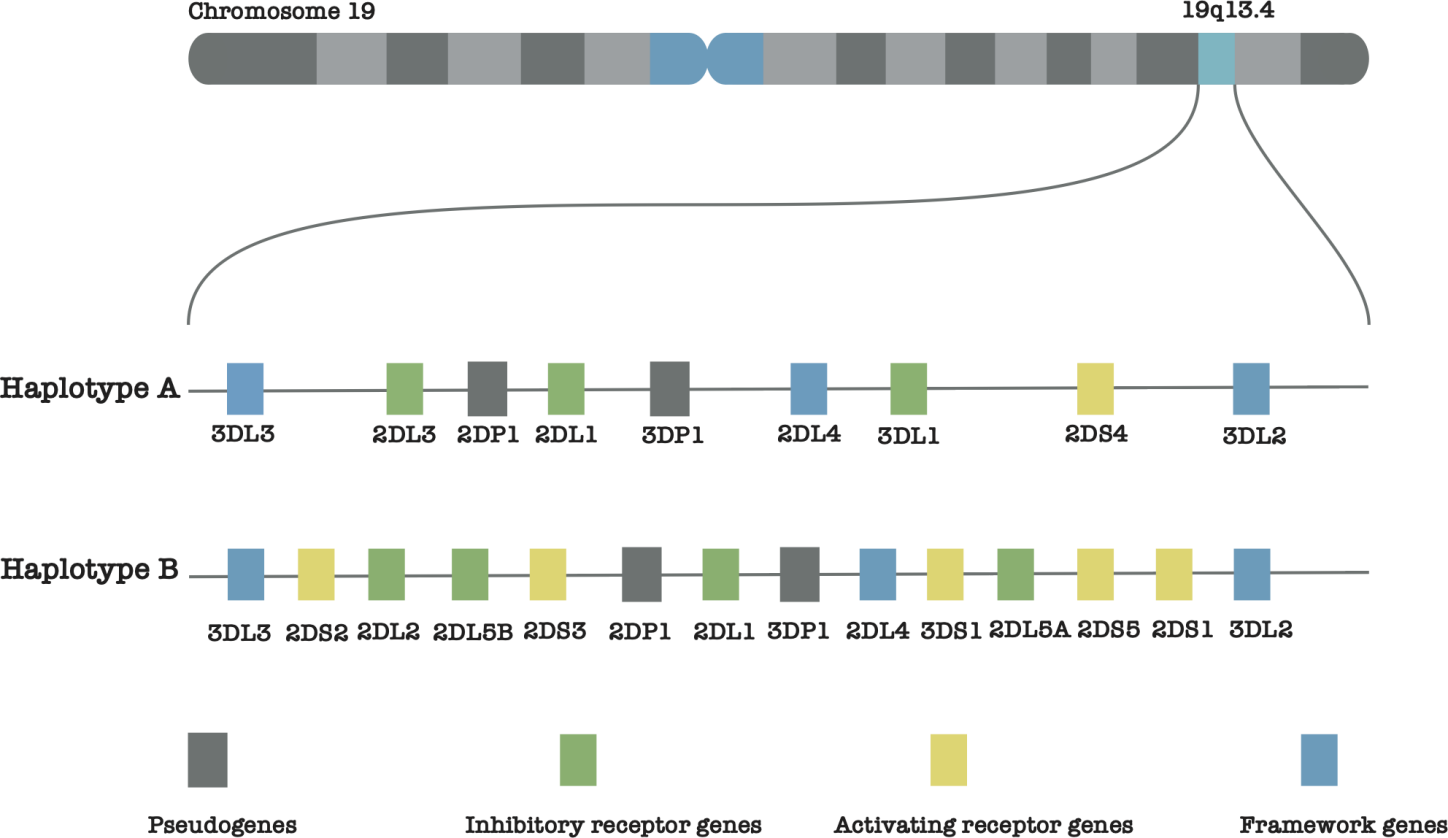
Illustration of the KIR gene positions on chromosome 19, showcasing the distinct structures of haplotype groups A and B.

The seventeen (17) KIR genes are named based on their extracellular Immunoglobulin-like (lg-like) domains (designated as 2D or 3D) and the lengths of their cytoplasmic tails (marked as L for long cytoplasmic tails, S for short cytoplasmic tails, and P for pseudogene). A general rule is that short-tailed KIRs are activating receptors, while long-tailed KIRs are inhibitory receptors. Based on these designations, the KIR genes can be categorized as follows: (a) six (6) genes with two domains and long cytoplasmic tails (*KIR2DL1*– *KIR2DL5B*), (b) five (5) genes with two domains and a short cytoplasmic tail (*KIR2DS1*–*KIR2DS5*), (c) three (3) genes with three domains and long tails (*KIR3DL1*–*KIR3DL3*), (d) one (1) *KIR3DS1* that is characterized by having three domains and a short tail, and (e) two (2) pseudogenes (*KIR2DP1* and *KIR3DP1*) 1. The whole-region KIR haplotypes are divided into two categories: group B (having one of *KIR2DL5*, *KIR2DS1*, *KIR2DS2*, *KIR2DS3*, *KIR2DS5* and *KIR3DS1*) and group A (having none of these genes) (7) (**Figure 1**). Finally, names of individual gene alleles, roughly follow the star-allele nomenclature used for gene annotation (9, 10), where each allele is assigned a number that indicates its function (8). The current known KIR alleles have been assembled and cataloged within the IPD-KIR database (11).

As different KIR alleles result in different immune responses, it is necessary to precisely genotype and phase KIR genes to better understand the role these genes play within the immune system. One cost-effective way of doing that is by using high-throughput sequencing (HTS) technologies that have been successfully used for large-scale genotyping (12). However, KIR genotyping cannot be easily done through the established HTS genotyping pipelines, such as GATK (13), primarily due to the high gene polymorphism of individual KIR genes. Not only KIR genes harbor many variants, but their alleles are defined by the whole gene phase—resolving this phase necessitates both variant calling and phasing. Another reason is that the copy number of each KIR gene varies significantly across individuals: while the presence of some genes is relatively uncommon (e.g., *KIR2DS3*), it is not rare to see some genes with large copy numbers (e.g., *KIR2DL4* or *KIR3DP1*), where each copy may have a different allele. Finally, the sequence contents of many KIR genes are mutually similar, which introduces high levels of ambiguity during the alignment of short reads to the KIR region. Such ambiguity is typically resolved in an arbitrary fashion, which produces incorrect alignments and, in turn, incorrect variant and allele calls. All these challenges are exacerbated by the reference genome itself: the latest canonical version of the human genome (GRCh38) does not include most of the KIR genes in the primary assembly and has no consistent reference model of the whole KIR region.

Some of these challenges have been previously encountered and addressed within the context of pharmacogene genotyping (14–16). Genes such as *CYP2D6*, *CYP2A6*, *CYP2C19*, and *SLCO1B1*, also exhibit high levels of polymorphism and are subject to various copy number and structural variation events, which makes them incompatible with the standard genotyping pipelines. Thus, many specialized genotyping tools specifically tailored for pharmacogenes have been recently proposed. Of these tools, Aldy (17), Cypiripi (18), PyPGx (19), StellarPGx (20), Stargazer (21), and Astrolabe (22). However, despite their success in the field of pharmacogenomics, these tools rely on the correct and precise alignments to the target genes to make correct allele calls and cannot handle complex regions such as KIR, where most of the read alignments are ambiguous. While one of these tools, Aldy 4 (23), provides some support for reads alignment within the *CYP2D* region, it cannot handle the scale and complexity of 17 KIR genes.

One genomic region that shares similar ambiguous alignment problems as KIR but has been successfully genotyped is the immunoglobulin heavy chain locus. The variable genes (IGHV) present in this locus are particularly challenging to genotype, with high polymorphism rate, copy number variants, structural variants, and homologous sequences (24). This problem has been addressed by the ImmunoTyper-SR tool (25, 26), which uses a combinatorial optimization approach to resolve read mapping and alignment ambiguities. However, while IGHV genes are numerous (∼ 120 functional and non-functional copies per chromosome), they are much shorter than KIR genes (∼ 280 bp vs 13.4 kbp), and the resulting difference in scale means that this approach cannot be utilized for KIR genotyping.

For these reasons, quite a few tools have been recently developed to assess the KIR region itself. The first group of tools solely focuses on annotating and genotyping KIR genes within whole genome assemblies and includes SKIRT (27), Immuannot (28) and BAKIR (29). These tools are, however, unable to handle sequencing data unless such data is assembled first, which cannot be done accurately with short-read sequencing data within complex regions such as KIR. The other group of tools, such as T1K (30), PING (31, 32), KASS (33), KPI (34) and KIR*IMP (35), are specifically designed for genotyping short-read sequencing data. Some tools, such as KPI, only handle gene-level identification and are unable to precisely call individual KIR alleles. KASS relies on *de novo* assembly of error-corrected sequences from PacBio’s long-read capture data that are annotated with KIR genes and exon/intron locations. Finally, tools such as PING, KIR*IMP, and T1K can identify individual alleles from the short-read sequencing data. PING utilizes *k*-mer fingerprinting to call individual alleles but is hard to run as it requires manual parameter estimation for each input cohort, and it also overlooks specific genes that are highly similar to each other, such as *KIR3DL1* and *KIR3DS1* (30). Another approach, KIR*IMP, relies on a statistical SNP imputation to call KIR alleles but is limited to high-quality SNPs and sufficiently large reference panels. Finally, T1K utilizes an expectation maximization strategy to rapidly identify KIR and HLA (Human Leukocyte Antigen) genotypes from sequencing data. While T1K offers speed and acceptable accuracy, it is currently not able to determine the copy number of KIR genes. It is also worth noting that many of these tools call alleles based solely on their sequence similarity to the reference KIR alleles and thus sometimes fail to distinguish alleles by their true functional impact, as sequence similarity is not a perfect proxy for functional characterization of sequences.

In order to address the outstanding challenges in analyzing and genotyping the KIR region, we introduce Geny, a *GENotYper for KIR genes*. This tool combines an expectation minimization-based filtering scheme with a combinatorial optimization approach in the form of integer linear programming [strategies inspired by Clever (36), OptiType (37) and our own pharmacogenomics tool Aldy (17)] to infer copy number and the exact allele of each present KIR gene copy. Furthermore, it can detect and leverage all variant types found in the KIR database and is able to distinguish between core, allele-defining variants that define the allele’s functionality and the silent variants that have no major impact on the overall functionality. We show that Geny is fast and achieves better precision and recall—up to 20%—over the existing KIR callers on both simulated and real datasets and that it provides significantly fewer miscalls than the other tools. As such, we hope that Geny lays the groundwork for precise KIR genotyping algorithms and that it will become a major part of future biomedical applications dealing with human immune system behavior.

## 2 Results

We assessed the performance of Geny and other major tools, T1K and PING, on two large datasets: simulated reads on top of fifty (50) completely assembled KIR regions from GenBank and on 40 whole genome Illuminasequenced HPRC samples. These samples represent a comprehensive benchmark due to their diversity and the presence of high-quality complete assemblies; as such, they became an safe choice for benchmarking the performance of KIR genotyping and annotating tools (28, 30).^^1^^

During the assessment of the performance of each tool, we computed the number of differences between the ground truth call and the inferred call, where the number of misses corresponds to the number of false positives and false negatives. We also provide the standard precision *P* (number of true positives divided by the number of true and false positives), recall *R* (number of true positives divided by the number of true positives and false negatives), and F1 scores $\left(2\frac{PR}{P+R}\right)$ for each tool. Each metric took into account the copy numbers as well. Note that we limited ourselves only to functional allele concordance (i.e., the first three-digit match of the allele name; thus, allele *0010101 is treated as *001) for consistency across the tools. Furthermore, we observed that many alleles in our datasets were novel and did not exactly match any of the alleles in the database, mostly due to the differing silent variants, and thus did not have an established name. Finally, note that some tools, such as PING, may output multiple possible solutions. In these cases, we selected the allele option that is closest to the ground truth and reported those as representative calls.

We compared Geny’s calls with T1K and PING, the only comparable KIR genotyping tools that provide allele-level genotype calls.^2^ However, we encounter several challenges when attempting to apply PING. Firstly, PING by default assumed samples from the same cohort. We also note that running PING required a lot of manual intervention and manual parameter inference, as the default set of parameters produced suboptimal results (see **Supplementary Materials** for details); on the other hand, both Geny and T1K required only input FASTQ or SAM/BAM/CRAM files to operate. PING also seemed to be extremely sensitive to the user-provided probe hit ratio thresholds for setting copy numbers of each gene. Finally, PING assumes that only one copy of *KIR3DL3* is present per haplotype to normalize the number of *k*-mer hits per gene in their copy number estimation stage. While they suggest using *KIR3DL2* to normalize the *k*-mer hit counts in case *KIR3DL3* is duplicated in a sample (39), it is unclear how to do so. As our simulated dataset did not satisfy the first and third criteria (cohort data and fixed *KIR3DL3* copy number), we were not able to apply PING to this dataset. On the HPRC dataset, we tried multiple versions of PING [e.g (32),^3^] under different set of parameters and selected the one that gave the best results (see **Appendix C** for details).

### 2.1 Ground truth annotations

The ground truth for each sample was obtained by analyzing the complete assemblies of the KIR region. The annotations were generated using the BAKIR tool (29) which was developed specifically for this application. Initially, the KIR allele database was aligned to the assembly with minimap2 (40), followed by the merging of all overlapping mappings to locate putative genes. The gene type was identified by selecting the gene with the highest number of alleles mapped. Subsequently, the wildtype of the identified gene was re-mapped to the putative gene sequence to refine its location again using minimap2. The refined putative gene sequence was then aligned with the wildtype sequence via global alignment using parasail (41), allowing for the calling of variants and identification of functional variants. The closest allele to the putative gene sequence was selected based on the allele with the lowest functional variant Jaccard distance relative to the wildtype sequence, employing non-functional Jaccard distance in cases of ties. In other words, we prefer alleles that preserve their functionality by (1) having all its core variants present (see Methods for the exact definitions) and (2) not introducing novel core variants. In the case of a tie, the allele with the smallest Jaccard distance from the wildtype sequence was selected.

In some instances, the second condition could not be fulfilled without breaking the first condition. Even if both conditions are satisfied, the assembled sequence might still differ from the KIR-IPD allele sequences due to the differences in silent variations. Both cases point out that the sequenced allele is novel and is not yet cataloged within the IPD-KIR database; in either case, we selected the database allele that is closest to the observed allele as the “ground truth” based on the above criteria.

Finally, we performed some manual interventions on top of KIR-Annotator calls. In the case of GenBank assemblies, we used the existing GenBank allele annotations where possible to cross-validate and correct our calls. We also manually checked the presence of exon 1 deletion within *KIR3DP1* region that KIR-Annotator was unable to detect on its own.

To minimize potential annotation-based biases, we also conducted cross-validations by comparing Geny’s performance against T1K and PING with ground truth annotations produced by SKIRT (27) and Immunanot (28) annotators on the HPRC assemblies.

### 2.2 Simulated data

We collected 50 complete assemblies of the KIR region from the GenBank (42), each corresponding to a distinct individual (**Supplementary Materials**). These assemblies cover a diverse set of KIR configurations, including cases with copy number variations, alleles from haplotype classes A and B, non-identified alleles and so on. Many of these assemblies already came with KIR allele annotations, which we used as the ground truth; the aforementioned annotation tools were used if the provided annotations were missing or out-of-date. Once these sequences were annotated, each was independently inserted within an assembly of chromosome 19 (at 54,724,235–54,867,216) to replace the KIR locus and create a synthetic KIR assembly sample. We then simulated perfect paired-end reads of size 100bp that cover this locus with the coverage of 20× for each synthetic assembly. In order to create diploid samples, representing the 2 copies of the KIR locus present in a human genome, we randomly selected pairs of synthetic assemblies and combined their simulated reads to create 21 synthetic diploid samples. The resulting samples encompassed all 17 KIR genes and pseudogenes, and contained 828 true alleles spread across these genes. The allele count for each gene is shown in **Table 1**.

**Table 1:** Comparison of Geny and T1K on simulated datasets from the 50 GenBank assemblies.

Gene	Total	Geny				T1K			
		Misses	Precision	Recall	F1	Misses	Precision	Recall	F1
*KIR2DL1*	66	**4**	**98.4%**	**95.4%**	**0.97**	10	91.0%	93.8%	0.92
*KIR2DL2*	29	**5**	**89.7%**	**92.9%**	**0.91**	6	89.3%	89.3%	0.89
*KIR2DL3*	55	**0**	**100.0%**	**100.0%**	**1.00**	5	96.3%	94.5%	0.95
*KIR2DL4*	81	**0**	**100.0%**	**100.0%**	**1.00**	5	**100.0%**	93.8%	0.97
*KIR2DL5A*	23	**3**	**88.5%**	**100.0%**	**0.94**	8	75.9%	95.7%	0.85
*KIR2DL5B*	13	**5**	**100.0%**	61.5%	**0.76**	70	13.6%	**100.0%**	0.24
*KIR2DP1*	68	**5**	**94.0%**	**98.4%**	**0.96**	9	91.0%	95.3%	0.93
*KIR2DS1*	26	**3**	**96.0%**	**92.3%**	**0.94**	4	95.8%	88.5%	0.92
*KIR2DS2*	28	**4**	**87.1%**	**100.0%**	**0.93**	8	80.0%	92.3%	0.86
*KIR2DS3*	16	**0**	**100.0%**	**100.0%**	**1.00**	40	27.8%	93.8%	0.43
*KIR2DS4*	62	**3**	**96.7%**	**98.3%**	**0.98**	14	83.3%	96.8%	0.90
*KIR2DS5*	18	**0**	**100.0%**	**100.0%**	**1.00**	**0**	**100.0%**	**100.0%**	1.00
*KIR3DL1*	61	**4**	**100.0%**	**93.4%**	**0.97**	11	88.5%	93.1%	0.91
*KIR3DL2*	87	**5**	**98.8%**	95.3%	**0.97**	33	69.7%	**95.8%**	0.81
*KIR3DL3*	82	**12**	**89.7%**	94.6%	**0.92**	33	68.0%	**98.6%**	0.80
*KIR3DP1*	89	**16**	**88.4%**	**92.7%**	**0.90**	26	84.1%	86.0%	0.85
*KIR3DS1*	24	**0**	**100.0%**	**100.0%**	**1.00**	6	87.0%	87.0%	0.87
All	828	**69**	**95.4%**	**96.0%**	**0.96**	288	75.5%	93.6%	0.84

Bold type indicates better results. Geny produces a significantly lower number of miscalls and outperforms T1K in all metrics, in some cases by a large margin (up to 20%).

Geny has more than 200 fewer misses over T1K, as can be seen in the results shown in **Table 1**. It also improves the precision by 20% and F1 score by 0.12. Geny outperformed T1K on all individual genes as well. We note that T1K had a high false positive rate on *KIR2DL5B* and *KIR2DS3* (70 and 40, respectively). It also assumed that the copy number of each gene does not exceed 2. Finally, it struggled with the KIR3DL gene family. However, its recall was competitively high, albeit slightly lower than Geny’s. We also note that Geny also had issues with the KIR3DL family, particularly with *KIR3DP1* and *KIR3DL3*, where it often completely missed the presence of these genes or exonic deletions that define some of the core *KIR3DP1* variants.

The cases where Geny misses the allele can be roughly explained as follows: (1) novel or non-standard alleles that have a non-standard combination of core variants or large exonic deletions and, as such, get filtered out; (2) an “extended” solution where the true allele mistakenly gets assigned an additional core variant due to incorrectly resolved cross-gene read alignments; and (3) copy number inconsistencies. While we plan to address cases (1) and (3) in the near future, we note that the second case is challenging to handle because the wrong solution can be explained by the observable reads based on the current model.

### 2.3 Real data

To evaluate the performance of Geny on real data, we conducted a comparative analysis using 40 whole genome samples sequenced by Illumina NovaSeq 6000 (read length 150bp) sourced from the 1000 Genomes Project (43). The ground truth for this comparison was derived from multi-model assemblies generated by the Human Pan Genome Consortium (44) and covers diverse ethnicities. As such, this dataset ensures that the evaluation reflects a highly realistic assessment of tools’ performance in real-world scenarios.

As shown in **Table 2**, Geny demonstrated strong performance relative to the other tools across various metrics, including precision, recall, F1 score, and miss rate. For instance, Geny missed nearly half as many alleles as T1K and less than a third as many as PING. It also performs well on individual KIR genes. The sole exceptions is the *KIR3DL3* gene, where PING produces the overall best results. In general, it is *KIR3DL3* and *KIR3DP1* that cause most of the trouble for T1K and Geny on this dataset; this is not surprising, as *KIR3DP1* has already been reported to pose significant challenges for correct genotyping (31). T1K also suffers from missing whole gene copies and wrong allele assignment (regardless if the overall copy number is below or above 2).^4^ In general, Geny’s misses follow the same patterns as observed on the simulation datasets. We note that many assemblies point to the existence of novel and uncatalogued KIR alleles; further work will be necessary to validate and catalog them correctly.

**Table 2:** Comparison of Geny, PING and T1K on 40 HPRC samples.

			Geny				T1K				PING		
Gene	Total	Misses	Precision	Recall	F1	Misses	Precision	Recall	F1	Misses	Precision	Recall	F1
*KIR2DL1*	68	**13**	85.9%	93.2%	**0.89**	20	**87.5%**	79.0%	0.83	46	50.0%	**93.5%**	0.65
*KIR2DL2*	19	**1**	**94.7%**	100.0%	**0.97**	2	89.5%	**100.0%**	0.94	5	93.3%	77.8%	0.85
*KIR2DL3*	60	**7**	93.0%	94.6%	**0.94**	12	**96.1%**	83.1%	0.89	15	90.0%	81.8%	0.86
*KIR2DL4*	78	**1**	**100.0%**	98.7%	**0.99**	24	88.5%	76.1%	0.82	14	82.1%	**100.0%**	0.90
*KIR2DL5A*	8	**3**	**72.7%**	**100.0%**	**0.84**	5	63.6%	87.5%	0.74	5	66.7%	75.0%	0.71
*KIR2DL5B*	14	**4**	**100.0%**	71.4%	0.83	6	68.8%	**91.7%**	0.79	3	91.7%	84.6%	**0.88**
*KIR2DP1*	69	**9**	93.8%	92.3%	**0.93**	13	**96.6%**	83.8%	0.90	35	50.0%	**97.1%**	0.66
*KIR2DS1*	14	**0**	**100.0%**	**100.0%**	**1.00**	2	**100.0%**	85.7%	0.92	12	**100.0%**	14.3%	0.25
*KIR2DS2*	17	**1**	94.1%	**100.0%**	**0.97**	1	**100.0%**	94.1%	**0.97**	3	82.4%	**100.0%**	0.90
*KIR2DS3*	8	**1**	**100.0%**	87.5%	0.93	2	87.5%	87.5%	0.88	0	**100.0%**	**100.0%**	**1.00**
*KIR2DS4*	66	**5**	95.4%	96.9%	**0.96**	12	**100.0%**	81.8%	0.90	9	86.6%	**100.0%**	0.93
*KIR2DS5*	14	**1**	**100.0%**	**92.9%**	**0.96**	4	85.7%	85.7%	0.86	3	84.6%	91.7%	0.88
*KIR3DL1*	66	**2**	**97.0%**	**100.0%**	**0.98**	6	95.2%	95.2%	0.95	36	69.9%	78.5%	0.74
*KIR3DL2*	78	8	**93.3%**	95.9%	0.95	30	74.6%	79.4%	0.77	7	91.0%	**100.0%**	**0.95**
*KIR3DL3*	80	**26**	70.1%	94.7%	0.81	36	57.1%	93.6%	0.71	16	**80.0%**	**100.0%**	**0.89**
*KIR3DP1*	78	**15**	86.3%	92.6%	**0.89**	27	**89.5%**	70.8%	0.79	16	79.5%	**100.0%**	0.89
*KIR3DS1*	12	**1**	92.3%	**100.0%**	**0.96**	2	**100.0%**	83.3%	0.91	2	**100.0%**	83.3%	0.91
All	749	**98**	**90.9%**	**95.3%**	**0.93**	204	86.0%	83.2%	0.85	227	76.3%	91.8%	0.83

Bold type indicates better results. Geny outperforms other tools in many of the metrics. The exceptions are the *KIR3DL2–3* and *KIR2DS3* genes, where PING does better than Geny and T1K.

To ensure that our findings are not biased by the selection of ground truth annotator, we also compared all tools against the ground truth annotations generated by SKIRT and Immunanot (**Appendices D**, **E**). In both cases, we observe the same trends and Geny still outperforms other tools. We note that these annotations consistently yield larger number of miscalls among all tools; for that reason, we opted to use BAKIR annotations as the “reference” as it better matches the overall consensus.

On a final note, we note that Geny quickly infers KIR genotypes: the current version of Geny typically takes from ten minutes to forty minutes to genotype all genes within an HPRC sample; in total, it needed around 18 hours to complete genotyping all samples (in sequential order). T1K typically needed two hours per HPRC sample (89 hours in total), while PING requires multiple samples at the same time and needed around 22 hours to genotype 40 HPRC samples. When possible, we used 8 threads to run a genotyping tool. All experiments were conducted on Linux instances with at least 92 CPU cores and at least 512 GB of RAM.

## 3 Discussion

The process of genotyping and phasing KIR genes is important for a deeper understanding of the innate immune system and its interactions. Here we have presented Geny, a new tool for identification of KIR alleles within high-throughput sequencing datasets. In our evaluations, Geny consistently outperformed the current state-of-the-art methods for KIR genotyping across many metrics on a diverse set of WGS samples. As we move toward tailored medical treatments, the accuracy of tools like Geny in identifying genes can shape the future of patient care.

There are still many areas left for improvement and further study. The next major step will be adding support for other sequencing technologies, such as long-read technologies (e.g., PacBio HiFi) or the targeted sequencing panels [e.g., TruSight One or Norman et al. (39)]. Limited support for whole-exome data (WES) is also considered; however, it should be noted that WES data is not well suited for genes and regions subject to various copy-number and structural events (45–47). Another is the detection of novel major alleles—functional alleles that have not been cataloged by the existing KIR databases. This also includes calling of fusion alleles that have been observed in the wild (48). We are also looking into incorporating more capable statistical models that can offer better performance over the current combination of the EM filtering algorithm and the ILP backbone. Finally, we are looking to incorporate HLA calling as well into the Geny pipeline to be able to study the complex interplay between KIR and HLA in immune responses (49).

Another major future task consists of a comprehensive evaluation of the quality of ground truth data and establishing systematic criteria for measuring the quality of genotyping, especially in the presence of novel alleles. The existing validated datasets, such as the IHIWS (50) or UCLA Cell Exchange program (51), primarily test for gene presence or absence data and often lack the accessible allele-level ground truth calls. These datasets also do not have public sequencing data available for download. Thus, they are still insufficient for precise benchmarking of KIR genotyping methods [a role that the GeT-RM project (52)] plays in the field of pharmacogenomics). While the GenBank samples and HPRC assemblies, together with the ensemble of KIR annotation tools, provide a solid basis for evaluation and may be sufficient for current needs, it is important to acknowledge that future work may require establishing consistent accuracy measurements and additional benchmarking and wet lab validation on a broader range of validated and accessible real-world samples.

Such an evaluation should also be accompanied by a systematic comparison of WGS-based genotyping strategies with the existing KIR-specific genotyping solutions (39, 53). These strategies are reported to generate more accurate genotyping calls than those observed for WGS data (especially in this study). Hence, studies akin to GeT-RM’s *CYP2C8*–*19* reconciliation (16) are sorely needed to understand better the baseline of the various KIR genotyping strategies (e.g., plain WGS versus the custom solutions) and reconcile various conflicting reports in the literature regarding the quality and accuracy of the proposed strategies.

## 4 Methods

### 4.1 Overview

The Geny pipeline consists of three major stages. The first stage reads the short-read HTS data from a FASTQ or a SAM/BAM file and computes all possible alignments to the reference KIR sequences for each read found in the sample. The second stage filters out unlikely KIR alleles and reads assignments by employing both deterministic and statistical criteria to reduce the overall search space and enhance the quality of the final calls. Finally, the third stage solves the integer linear programming (ILP) model that determines the correct copy number and the exact allele (phase) of each present KIR gene.

### 4.2 Preliminaries: notation and database preparation

Each Geny stage requires the annotated KIR allele database. We use the latest version of the IPD-KIR database (11) (v2.12.0) that contains the allelic sequences of currently known KIR alleles, as well as the associated allele names that characterize their functionality. Each name is a set of at most seven (7) digits, where the first three digits indicate the allele functionality (typically defined by the non-synonymous exonic changes), the next two digits indicate the synonymous exonic changes, and the last two digits indicate all other changes (2). In the rest of the section, we will utilize the terminology from pharmacogenomics (23) and will refer to alleles with different functionality as *major alleles*. For example, KIR2DL2*0010101 and KIR2DL2*0010105 both encode the same major allele (KIR2DL2*001) and thus the same protein, while only differing by a couple of non-exonic variants.

Determination of the functional behavior of present KIR alleles (in other words, *major allele* calling) is the key aspect of the KIR genotyping process. The functional behavior is, in turn, defined by the set of *core variants*: variants that distinguish the functionality of a given allele from the other alleles. While these variants are typically functional (including both SNPs and indels of various sizes), they can also include UTR variants, whole exon deletions, and other variants that affect gene expression. All other variants that do not impact the allele’s function are called *silent variants*.

Unlike pharmacogenomics databases such as PharmVar, the KIR-IPD database contains only the allelic sequences for each allele and does not provide a list of core variants that differentiate those alleles from the reference (wildtype) allele (typically denoted as *001 or *0010101). Most of the available annotation tools, even when annotating complete assemblies, only rely on a simple edit distance score to compare allele sequences and oftentimes fail at properly determining the correct allele calls because they do not distinguish between core (functional) and silent variants. For example, KIR2DS1*011 allele is defined by the core c.5812 G*>*A functional variant that distinguishes its functionality from KIR2DS1*002 (the reference allele). While many other variants also distinguish *011’s sequence from *002’s, they are either silent or intronic and can be ignored when testing for the presence of *011. However, if all variants are considered the same (as they are in edit distance calculation), the lack of a few silent variants will overcome the concordance of a single core variant and might result in the wrong major allele assignment. To avoid this issue, we developed a PharmVar-like allele database for each KIR gene by aligning each allele sequence from the KIR-IPD database to the reference allele with parasail (41) and calculating the list of core variants that define each allele. We also established the complete genomic sequence for each allele: while the IPD-KIR database contains complete sequences for most of its alleles, there are cases where it only provides the coding sequence or a small exonic part that differentiates the allele from the reference allele. Finally, based on the existing literature [e.g., (54, 55)] and GenBank annotations, we constructed a KIR locus reference sequence that contains all 17 KIR genes and used it during the alignment step.

Formally, the final Geny database contains a set of KIR genes $G=\left\{G_{1},\ldots ,G_{17}\right\}$ . Each gene $G_{g}\in G$ harbors a list of variants ${ℳ}_{g}\;=\left\{m_{g,1},m_{g,2},\ldots \right\}$ and a set of alleles $A_{g}=\left\{A_{g,}1,A_{g,}2,\ldots \right\}$ . The allele $A_{g,}1$ is considered to be *reference allele*. Let $A=\cup_{g}A_{g}$ . Each allele $A_{g,i}$ is in turn defined by a variants ${ℳ}_{g,i}\subseteq {ℳ}_{g}$ . Each variant $m_{g,j}\in {ℳ}_{g}$ is a tuple (*l_g,j_,o_g,j_*) containing its location *l_g,j_* in the reference allele $A_{g,}1$ and an operation *o_g,j_* (SNP or an indel). For example, the previously mentioned c.5812 G*>*A is encoded as (5812*,GA*). A mutation $m_{g,j}\in {ℳ}_{g}$ is a core variant iff core(*m_g,j_*) = 1. A location *l* in gene $G_{g}$ is called *core location* if there is allele $A_{g,i}\in A_{g}$ that has a core variant at location *l*. Finally, each allele $A_{g,i}$ is assigned **a** *_g,i_* that corresponds to its genomic sequence. **a** *_g,i_* [*l*] indicates the *l*-th position in such sequence.

### 4.3 Stage 1: alignment

The first step of Geny pipeline aligns the input reads $ℛ=\left\{r_{1},r_{2},\ldots \right\}$ to the allele sequences **a** *_g,i_*. Because many reads in the KIR region can be aligned to many different alleles across many genes, Geny needs to compute all alignments from each read to each KIR allele from the database. We use minimap2 (40) in all-to-all mode that can handle short-reads (–dual=no -P –secondary=yes) to achieve this. Following the alignment, Geny discards all alignments that contradict the core variants for each allele, severely clipped alignments, and those that have a low alignment score. Finally, we end up with a set of alignments *H_k_* ={*h_k,g_*_1_ *_,i_*_1_ *,h_k,g_*_2_ *_,i_*_2_ *,…*} for each read $r_{k}\in ℛ$ . Each alignment indicates the target allele sequence **a** *_g,i_*, as well as the location on it and the edit operation needed to align the read to it.

In order to be able to determine the copy number of each KIR gene, we also align input reads to a copy number-neutral region in the genome. By default, we use copy number-neutral *COMT* gene region; other choices can be provided by the end user. Alignment to the copy number-neutral region provides the expected coverage of the sequencing data that is used later to call copy numbers and alleles.

### 4.4 Stage 2: filtering

The large number of the KIR alleles—the current version of the KIR-IPD database contains more than 1,500 known alleles among 17 KIR genes—adversely impacts the search space of the subsequent combinatorial optimization step. Therefore, Geny attempts to limit the number of valid alleles by filtering out those that are unlikely to occur based on the alignment data.

In the first pass, Geny selects only those alleles whose core variants are covered by a sufficient number of reads that are considered. By default, we set the minimum allowed read coverage to 3. We also need to ensure that alleles that do not harbor a core variant at a core variant site still have sufficient coverage at that site to be considered.

#### 4.4.1 Landmark generation

In addition to filtering out the unlikely alleles, it is also important to filter out the spurious read alignments. Ideally, we would like to consider only a small set of alignments that map to the core locations of each allele remaining after the previous filtering and discard other reads. However, as many reads that map to the core locations also map to regions within other genes that contain no core locations specific to that gene, we need to extend the set of core locations to also include other locations that “mirror” the core locations in other genes. Thus, we introduce the concept of *landmark locations* that are projections of the valid core locations to all alleles (of any gene) that, despite not harboring core variants, still “catch” the reads that cover core variants in other alleles. The objective of landmarks is to provide the opportunity for the reads to be assigned to non-variant harboring alleles.

**Figure 2:**
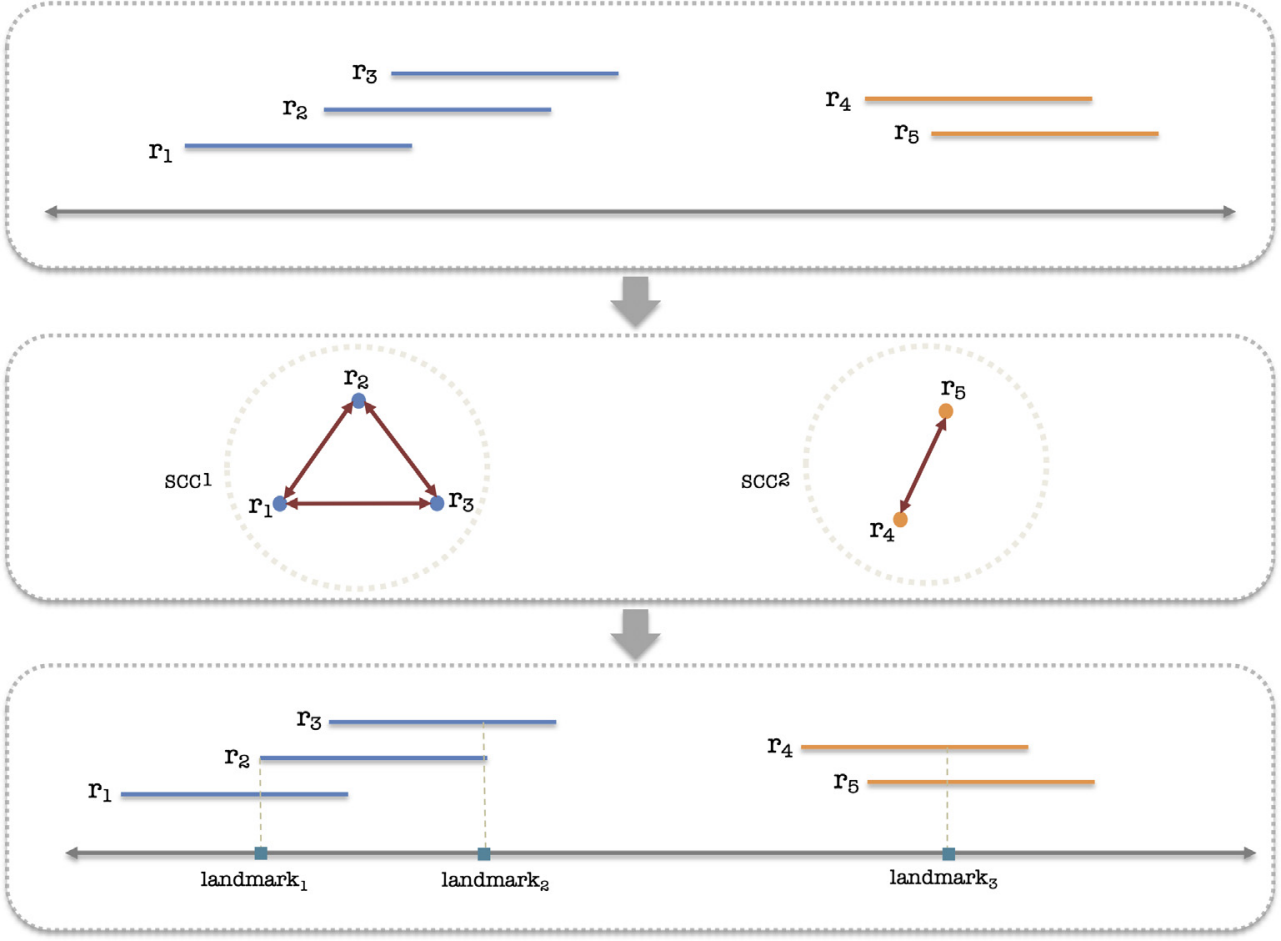
Diagram illustrating the process of landmark generation. Initially, all valid reads are collected as input. These reads are then utilized to construct a graph that represents their overlap. Then, we identify strongly connected components (SCCs) of that graph, which represent groups of reads that continually cover a region by overlapping each other. In the concluding step, we infer the landmarks based on the SCCs of the graph concerning each candidate allele.

To infer landmarks, we construct an overlap graph *G_g,i_* for each candidate allele $A_{g,i}\in A_{g}$ to capture the relationships between the alignments that cover the variants ${ℳ}_{g,i}$ (**Figure 2**). We consider all alignments *h_k,g,i_* which either cover a core location or for which there is another alignment $h_{k,g^{\prime },i^{\prime }}$ in another gene or allele that covers a core location. Each such alignment *h_k,g,i_* corresponds to a node in the graph. A graph edge is created between two alignments if they overlap on **a** *_g,i_*. Constructing the overlap graph enables us to identify the *landmark regions* in **a** *_g,i_* that harbor “interesting” reads; those regions can be found by finding the strongly connected components (SCCs) within each *G_g,i_*. Once landmark regions are identified, we establish landmark locations *L_g_* = *l*_1_ *,l*_2_ *,…* for each gene $G_{g}$ by augmenting the set of gene’s core locations with an appropriate number of other positions in $G_{g}$ so that each alignment that covers a landmark region also covers at least one landmark position. Finally, we select all alignments that are covered by a landmark position and discard the others.

#### 4.4.2 Candidate allele selection

After obtaining a set of valid alleles and reads, Geny further filters the set of valid alleles through the Expectation Maximization (EM) algorithm (56) by identifying alleles with lower densities in the input sample, thus reducing the solution space for the final solver and improving specificity (57). The EM algorithm, in a setting where the input data is partially known and the parameters of the distribution function (model) that generated the data are unknown, iteratively estimates the parameters of the model to maximize the likelihood of the observed data. We perform maximum likelihood estimation on the abundance of each allele and the sequencing error rate in a similar fashion as in (58).

Let *ϕ* denote the abundance of each of the n=|A| candidate alleles, ℒ(θ) the log-likelihood of the read set ℛ consisting of m=|ℛ| total reads given the parameter $\theta ,Z_{k}$ the latent variable representing the allele which generates *r_k_*, and *ϕ_i_* the density of allele $A_{i}\in A$ . Let the sequencing error rate be *ϵ*. Consider there are $p_{k}^{i}$ matching bases for mapping read $r_{k}\in ℛ$ on $A_{i}\in A$ with allele length *l_i_*. Let $l_{k}^{i}$ be the number of bases read *r_k_* maps on allele $A_{i}$ . To account for multiple possible alignments of a read within a single allele, we define $m_{k}^{i}$ as the count of valid alignments of *r_k_* in $A_{i}$ . Then:


(1) $$P\;\left(r_{k}\;|\;Z_{k}=i\right)=\frac{m_{k}^{i}\varepsilon^{\left(l_{k}^{i}-p_{k}^{i}\right)}{\left(1-\varepsilon \right)}^{p_{k}^{i}}}{l_{i}}.$$

We define the log-likelihood ℒ(θ) as follows:


(2) $$ℒ\left(\theta \right)=\text{log }P\;(ℛ\;|\;\theta )=\sum_{k}\text{log }\sum_{i}P\;\left(r_{k}\;|\;Z_{k}=i\right)\;P\;\left(Z_{k}=i;\;\theta \right).$$

Following this, we obtain the EM update steps for parameters *ϕ* and *ϵ* as follows (see **Supplementary Materials** for details). Let $\mu_{k}^{i}$ = *P* (Z*_k_* = *i* | *r_k_*). Then:


$$\phi_{i}^{\left(t+1\right)}=\frac{\sum_{k}\mu_{k}^{i}}{m},$$

$$\epsilon^{\left(t+1\right)}=\frac{\sum_{k}\sum_{i}\mu_{k}^{i}\left(l_{k}^{i}-p_{k}^{i}\right)}{\sum_{k}\sum_{i}\mu_{k}^{i}l_{k}^{i}},\text{ and }$$

$$\theta^{\left(t+1\right)}=\left(\phi_{i}^{\left(t+1\right)};\varepsilon^{\left(t+1\right)}\right).$$

Once the updated parameter values are obtained, we select all alleles associated with components of non-trivial presence, where *ϕ > γ*, for further refinement in the final stage and discard the others. Unlike other methods that use EM to select the final solution, we set *γ* to a small value (10^−3^) and only use this step *to filter out unlikely candidates*. This ensures that Geny avoids the common problem with EM-based methods, where the final solution ends up being a local maximum that is not relevant to the true call.

### 4.5 Stage 3: allele calling

For the final phase of allele calling, we aim to apply the Integer Linear Programming model (ILP),which has been shown to be effective on other highly polymorphic immune genes such as HLA (37), to assign each read to a proper KIR allele that passed the previous filtering stages and select the true alleles present in the sample. The problem is set as follows.

For each read $r_{k}\in ℛ$ and allele $A_{g,i}\in A$ , we introduce a binary variable *V_k,g,i_* that is set if and only if *h_k,g,i_* is the best alignment among all candidate alignments *H_k_* ^5^. In other words, *V_k,g,i_* indicates that the read *r_k_* is assigned to $A_{g,i}$ . We also allow the possibility of dropping reads—i.e., not assigning it to either of the alleles—by introducing the variable $D_{k}=1-\sum_{g,i}V_{k,g,i}$ . We associate a read drop cost *β* (0.08) with dropping each read. Let us also introduce an integer variable $A_{g,i}$ that is set to the number of times allele $A_{g,i}$ is selected in the final solution. Let the constant *ξ* denote the expected coverage of a single allele copy (determined via copy number-neutral region in Stage 1). Denote the minimum and maximum average percent of coverage with respect to expected coverage over landmarks of $A_{g,i}$ to be *ε* (0.5 by default) and *ϕ* (1.5 by default), respectively.

An integer linear program that selects a set of alleles among A and assigns the reads to them to minimize the difference between the observed coverage and the selected one can be formulated as follows:


$$\text{Minimize}:\;\sum_{g,i}\sum_{l\in L_{g}}\left|\sum_{k:h_{k,g,i}\text{ covers }l}V_{k,g,i}-\xi A_{g,i}\right|+\gamma \sum_{g,i}A_{g,i}+\beta \sum_{k}D_{k}$$

$$\text{Subject to}:\;\phi A_{g,i}\xi \left|L_{g}\right|\geq \sum_{l\in L_{g}}\sum_{k:h_{k,g,i}\text{ covers }l}V_{k,g,i}\geq \epsilon A_{g,i}\xi \left|L_{g}\right|,\;\forall k\;\forall g,i$$

$$A_{g,i}\geq V_{k,g,i},\;\forall k\;\forall g,i$$

$$\sum_{k}V_{k,g,i}\geq A_{g,i},\;\forall g,i$$

*L_g_* is a set of landmarks for the gene *G*. A selection cost *γ* is associated with each selected allele, and a read drop cost *β* for discarding each read.

By formulating the problem as an ILP and minimizing the total absolute coverage error, we effectively optimize the assignment of reads to alleles, resulting in more accurate and reliable allele identification. We employed Gurobi as a reliable tool for solving ILP problems in an efficient manner (59). Note that, unlike models used in pharmacogenomics [e.g., Aldy 4 (23)], this model performs read selection and is thus an order of magnitude more complex than the previous models. Currently, the model deploys tens of thousands of binary variables and hundreds of continuous variables.

## Data Availability

The original contributions presented in the study are included in the article/**Supplementary Material**. Further inquiries can be directed to the corresponding authors. Geny is available at https://github.com/0xTCG/geny and also uploaded as a Supplemental Code. The experimental procedure and results are available at https://github.com/0xTCG/geny/tree/master/paper and are also uploaded as Supplemental Notebook and Supplemental Experiments, respectively.

## Footnotes

1 Note that the other data sources from the literature either (i) do not have public WGS data available, (ii) are not assembled, or (iii) its KIR allele calls are not independently validated.

2 We also attempted to evaluate Graph-KIR (38); however, we were unable to get satisfactory results on our datasets. Hence, we excluded this tool from the comparison.

3 The latest Singluarity workflow from the https://github.com/Hollenbach-lab/PING .

4 We also ran T1K with the recently developed t1k-copynumber.py wrapper that provides limited support for copy-number calling. We observed no significant improvements; see **Appendix F** for more details.

5 For the sake of explanation and to avoid notation abuse, we assume that each read can only be aligned to a single location within a given allele; in practice, this is not true but the overall model applies to this case as well.

## Appendix A EM algorithm

Given the likelihood function with respect to parameters *θ* as:

$$ℒ\left(\theta \right)=\text{log }P(ℛ|\theta )=\sum_{k=1}^{m}\text{log }\sum_{i=1}^{n}P(r_{k}|Z_{k}=i)P\left(Z_{k}=i;\theta \right).$$

We assume each read *r_k_* is uniformly sampled from each allele $A_{i}$ with a length of allele *l_i_*, and each base of the read are independently generated with a sequencing error rate *ϵ*, and there are $p_{k}^{i}$ matching bases mapping *r_k_* on $A_{i}$ . Considering multi-mapping of read *r_k_* within allele $A_{i}$ , assume read *r_k_* maps to $l_{k}^{i}$ positions on allele $A_{i}$ , and $m_{k}^{i}$ be number of times *r_k_* aligns on $A_{i}$ we have:

(1)
$$P(r_{k}|Z_{k}=i;\theta )=\frac{m_{k}^{i}\varepsilon^{\left(l_{k}^{i}-p_{k}^{i}\right)}{\left(1-\varepsilon \right)}^{p_{k}^{i}}}{l_{i}}.$$

Let

$$Q(\theta |\theta^{t})=E_{Z|ℛ;\theta^{t}}\left[\text{log }ℒ\left(ℛ,Z;\theta \right)\right]=\sum_{k=1}^{m}\sum_{i=1}^{n}\;P\;\left(Z_{k}=i|r_{k},\theta^{t}\right)\;\text{log }P\left(r_{k},Z_{k}=i;\theta \right).$$

For each *i, j* we compute:

(2)
$$\mu_{k}^{i}=P(Z_{k}=i\;|\;r_{k})=\frac{P\left(Z_{k}=i\right)P(r_{k}|Z_{k}=i)}{\sum_{s=1}^{n}P\left(Z_{k}=s\right)P(r_{k}|Z_{k}=s)}.$$

To maximize *Q*(*θ*|*θ^t^*), we construct a Lagrangian:

(3)
$$\begin{array}{c} L\left(\phi ,\varepsilon ,\beta \right)=\sum_{k=1}^{m}\sum_{i=1}^{n}\mu_{k}^{i}\text{log }\left(\frac{m_{k}^{i}\varepsilon^{\left(l_{k}^{i}-p_{k}^{i}\right)}{\left(1-\varepsilon \right)}^{p_{k}^{i}}}{l_{i}}\phi_{i}\right)\\ +\beta \left(\sum_{i=1}^{n}\phi_{i}-1\right). \end{array}$$

To update *ε*, by KKT condition, we set:

(4)
$$\frac{\partial L\left(\phi ,\varepsilon ,\beta \right)}{\partial \varepsilon }=0.$$

Then:

(5)
$$\frac{\partial \sum_{k=1}^{m}\sum_{i=1}^{n}\mu_{k}^{i}log\;\left(\frac{m_{k}^{i}\varepsilon^{\left(l_{k}^{i}-p_{k}^{i}\right)}{\left(1-\varepsilon \right)}^{p_{k}^{i}}}{l_{i}}\phi_{i}\right)+\beta \left(\sum_{i=1}^{n}\phi_{i}-1\right)}{\partial \varepsilon }=0$$

$$\sum_{k=1}^{m}\sum_{i=1}^{n}\mu_{k}^{i}\frac{\partial log\;\left(\frac{m_{k}^{i}\varepsilon^{\left(l_{k}^{i}-p_{k}^{i}\right)}{\left(1-\varepsilon \right)}^{p_{k}^{i}}}{l_{i}}\phi_{i}\right)}{\partial \varepsilon }=0$$

$$\sum_{k=1}^{m}\sum_{i=1}^{n}\mu_{k}^{i}\left(l_{k}^{i}-p_{k}^{i}\right)-\varepsilon \sum_{k=1}^{m}\sum_{i=1}^{n}\mu_{k}^{i}l_{k}^{i}=0.$$

Thus:

(6)
$$\varepsilon^{t+1}=\frac{\sum_{k=1}^{m}\sum_{i=1}^{n}\mu_{k}^{i}\left(l_{k}^{i}-p_{k}^{i}\right)}{\sum_{k=1}^{m}\sum_{i=1}^{n}\mu_{k}^{i}l_{k}^{i}}$$

To update *ϕ_i_*, set:

(7)
$$\frac{\partial L\left(\phi ,\varepsilon ,\beta \right)}{\partial \phi_{i}}=0.$$

Thus:

(8)
$$\frac{\partial \sum_{k=1}^{m}\sum_{i=1}^{n}\mu_{k}^{i}log\;\left(\frac{m_{k}^{i}\varepsilon^{\left(l_{k}^{i}-p_{k}^{i}\right)}{\left(1-\varepsilon \right)}^{p_{k}^{i}}}{l_{i}}\phi_{i}\right)+\beta \left(\sum_{s=1}^{n}\phi_{s}-1\right)}{\partial \phi_{i}}=0$$

We have:

(9)
$$\phi_{i}^{t+1}=\frac{-\sum_{k=1}^{m}\mu_{k}^{i}}{\beta }.$$

Since $\sum_{i=1}^{n}\phi_{i}^{t+1}=1$ , we have $\sum_{i=1}^{n}\frac{-\sum_{k=1}^{m}\mu_{k}^{i}}{\beta }=1$ , thus:

(10)
$$\beta =-\sum_{k=1}^{m}\sum_{i=1}^{n}\mu_{k}^{i}=-m$$

Applying (10) to (9), we get:

(11)
$$\phi_{i}^{t+1}=\frac{\sum_{k=1}^{m}\mu_{k}^{i}}{m}.$$

## Appendix B Accession numbers

GenBank IDs that contain complete KIR region assemblies and were used for simulations:

**Table:** GenBank IDs of the assemblies with the complete KIR region used in the experimental section.

• NT_113949.2 • NT_187636.1 • NT_187637.1 • NT_187638.1 • NT_187639.1 • NT_187640.1 • NT_187641.1 • NT_187642.1 • NT_187643.1 • NT_187644.1 • NT_187645.1 • NT_187668.1 • NT_187669.1 • NT_187670.1 • NT_187671.1 • NT_187672.1 • NT_187673.1	• NT_187674.1 • NT_187675.1 • NT_187676.1 • NT_187677.1 • NT_187683.1 • NT_187684.1 • NT_187685.1 • NT_187686.1 • NT_187687.1 • NT_187693.1 • NW_003571054.1 • NW_003571055.2 • NW_003571056.2 • NW_003571057.2 • NW_003571058.2 • NW_003571059.2 • NW_003571060.1	• NW_003571061.2 • NW_016107300.1 • NW_016107301.1 • NW_016107302.1 • NW_016107303.1 • NW_016107304.1 • NW_016107305.1 • NW_016107306.1 • NW_016107307.1 • NW_016107308.1 • NW_016107309.1 • NW_016107310.1 • NW_016107311.1 • NW_016107312.1 • NW_016107313.1 • NW_016107314.1

Accession numbers for 40 HPRC samples used in the experimental section:

**Table:** Accession IDs of the HPRC samples used in the experimental section.

• HG00438 • HG00621 • HG00673 • HG00733	• HG00735 • HG00741 • HG01071 • HG01106	• HG01109 • HG01175 • HG01243 • HG01258
• HG01358 • HG01361 • HG01891 • HG01928 • HG01952 • HG01978 • HG02055 • HG02080 • HG02145 • HG02148	• HG02257 • HG02572 • HG02622 • HG02630 • HG02717 • HG02723 • HG02818 • HG02886 • HG03098 • HG03453	• HG03486 • HG03492 • HG03516 • HG03540 • HG03579 • NA18906 • NA19240 • NA20129
