# Supplementary material for: Geny: a genotyping tool for allelic decomposition of killer cell immunoglobulin-like receptor genes
Source: Front Immunol. 2024 Dec 23;15:1494995. doi: 10.3389/fimmu.2024.1494995 (PMC11701374; doi:10.3389/fimmu.2024.1494995)
Supplement: Supplementary file 1 [file Table1.pdf]

Appendix C Running PING on the Validation Dataset

We used the version of PING from `https://github.com/wesleymarin/PING/tree/wgs_snakemake`, as mentioned in [32], since we wanted to run it on WGS data. We extracted paired-end FASTQ files from the 40 HPRC WGS BAMs which were fed to PING. The tool was given 32 GB of memory and had access to 16 CPUs; scratch space was 100 GB.

We used the following probe hit ratio thresholds (Supplementary Table 1) for the copy numbers of each KIR gene, based on the initial plots generated by PING. The table is in the same format as the example threshold file (`manualCopyThresholds_example.csv`) that was packaged with the tool.

	**0-1**	**1-2**	**2-3**	**3-4**	**4-5**	**5-6**
KIR3DP1	0.15	0.35	0.6	0.8	NA	NA
KIR2DS5	0.2	0.6	1.1	NA	NA	NA
KIR2DL3	0.2	0.6	1	NA	NA	NA
KIR2DP1	0.3	0.6	1.2	NA	NA	NA
KIR2DS3	0.2	0.7	1.2	NA	NA	NA
KIR2DS2	0.18	0.5	0.8	NA	NA	NA
KIR2DL4	0.2	0.7	1.2	1.7	NA	NA
KIR3DL3	0	0	NA	NA	NA	NA
KIR3DL1	0.2	0.6	0.7	1	NA	NA
KIR3DS1	0.2	0.6	0.9	NA	NA	NA
KIR2DL2	0.15	0.28	0.45	1.1	NA	NA
KIR3DL2	0.2	1.1	1.6	NA	NA	NA
KIR2DS4	0.2	0.8	1.2	NA	NA	NA
KIR2DL1	0.2	0.47	0.59	0.8	1.2	NA
KIR2DS1	0.15	0.4	NA	NA	NA	NA
KIR2DL5	0.15	0.5	0.8	1.1	NA	NA

Supplementary Table 1: Thresholds for probe hit ratios (mean number of probe hits per target KIR gene divided by the mean number of *KIR3DL3* probe hits) used to generate validation results on PING.

The latest Singlularity-based PING distribution [32] is not well-suited for WGS data; on HPRC data, it achieved F1 score of 0.35 (neither precision nor recall exceeded 40%). For that reason, we opted not to present its detailed performance.

Appendix D Comparison With SKIRT Annotations

The following table summarizes the results over 40 HPRC samples using the SKIRT [27] annotations, Geny shows the overall best results on all metrics.

		Geny				T1K				PING			
Gene	Total	Misses	Precision	Recall	F1	Misses	Precision	Recall	F1	Misses	Precision	Recall	F1
KIR2DL1	68	**14**	84.4%	93.1%	**0.89**	20	**87.5%**	79.0%	0.83	47	48.8%	**93.3%**	0.64
KIR2DL2	19	2	89.5%	**100.0%**	0.94	**1**	94.7%	**100.0%**	**0.97**	4	**100.0%**	78.9%	0.88
KIR2DL3	61	**9**	91.2%	**92.9%**	**0.92**	16	88.2%	81.8%	0.85	12	**98.0%**	81.7%	0.89
KIR2DL4	78	**1**	**100.0%**	98.7%	**0.99**	24	88.5%	76.1%	0.82	14	82.1%	**100.0%**	0.90
KIR2DL5A	8	**3**	**72.7%**	**100.0%**	**0.84**	5	63.6%	87.5%	0.74	5	66.7%	75.0%	0.71
KIR2DL5B	14	4	**100.0%**	71.4%	0.83	6	68.8%	**91.7%**	0.79	**3**	91.7%	84.6%	**0.88**
KIR2DP1	68	**10**	**90.6%**	93.5%	**0.92**	23	79.7%	81.0%	0.80	36	47.1%	**100.0%**	0.64
KIR2DS1	14	**2**	85.7%	**100.0%**	**0.92**	4	83.3%	83.3%	0.83	12	**100.0%**	14.3%	0.25
KIR2DS2	17	**1**	**94.1%**	**100.0%**	**0.97**	3	87.5%	93.3%	0.90	3	82.4%	**100.0%**	0.90
KIR2DS3	8	1	**100.0%**	87.5%	0.93	2	87.5%	87.5%	0.88	**0**	**100.0%**	**100.0%**	**1.00**
KIR2DS4	67	**4**	96.9%	96.9%	**0.97**	13	**100.0%**	80.6%	0.89	8	88.1%	**100.0%**	0.94
KIR2DS5	14	4	76.9%	90.9%	0.83	7	64.3%	81.8%	0.72	**1**	**100.0%**	**92.9%**	**0.96**
KIR3DL1	66	**2**	**97.0%**	**100.0%**	**0.98**	6	95.2%	**95.2%**	0.95	36	69.9%	78.5%	0.74
KIR3DL2	79	10	92.0%	94.5%	0.93	31	73.1%	79.0%	0.76	**7**	**92.3%**	**98.6%**	**0.95**
KIR3DL3	80	26	70.1%	94.7%	0.81	31	63.6%	94.2%	0.76	**9**	**88.8%**	**100.0%**	**0.94**
KIR3DP1	78	18	82.2%	92.3%	0.87	29	**86.0%**	70.0%	0.77	**17**	78.2%	**100.0%**	**0.88**
KIR3DS1	12	**1**	92.3%	**100.0%**	**0.96**	2	**100.0%**	83.3%	0.91	2	**100.0%**	83.3%	0.91
All	751	**112**	**89.1%**	**95.1%**	**0.92**	223	83.3%	82.6%	0.83	216	78.0%	91.8%	0.84

Supplementary Table 2: Comparison of Geny, PING and T1K on 40 HPRC samples, using SKIRT [27] annotations. Bold type indicates better results.

Appendix E Comparison With Immuannot Annotations

		Geny				T1K				PING			
Gene	Total	Misses	Precision	Recall	F1	Misses	Precision	Recall	F1	Misses	Precision	Recall	F1
KIR2DL1	68	**13**	85.9%	93.2%	**0.89**	19	**89.3%**	79.4%	0.84	46	50.0%	**93.5%**	0.65
KIR2DL2	19	2	89.5%	**100.0%**	0.94	**1**	94.7%	**100.0%**	**0.97**	4	**100.0%**	78.9%	0.88
KIR2DL3	60	**8**	91.2%	**94.5%**	**0.93**	17	86.3%	81.5%	0.84	11	**98.0%**	83.1%	0.90
KIR2DL4	74	**5**	**94.8%**	98.6%	**0.97**	23	83.6%	79.7%	0.82	17	78.2%	**100.0%**	0.88
KIR2DL5A	8	**3**	**72.7%**	**100.0%**	**0.84**	5	63.6%	87.5%	0.74	5	66.7%	75.0%	0.71
KIR2DL5B	14	4	**100.0%**	71.4%	0.83	6	68.8%	**91.7%**	0.79	**3**	91.7%	84.6%	**0.88**
KIR2DP1	69	**11**	**90.6%**	92.1%	**0.91**	22	81.4%	81.4%	0.81	37	47.1%	**97.0%**	0.63
KIR2DS1	14	**2**	85.7%	**100.0%**	**0.92**	4	83.3%	83.3%	0.83	12	**100.0%**	14.3%	0.25
KIR2DS2	17	**1**	**94.1%**	**100.0%**	**0.97**	3	87.5%	93.3%	0.90	3	82.4%	**100.0%**	0.90
KIR2DS3	8	1	**100.0%**	87.5%	0.93	2	87.5%	87.5%	0.88	**0**	**100.0%**	**100.0%**	**1.00**
KIR2DS4	65	**6**	93.8%	96.8%	**0.95**	12	**98.1%**	82.8%	0.90	9	86.6%	**100.0%**	0.93
KIR2DS5	14	4	76.9%	90.9%	0.83	7	64.3%	81.8%	0.72	**1**	**100.0%**	**92.9%**	**0.96**
KIR3DL1	66	**2**	**97.0%**	**100.0%**	**0.98**	6	95.2%	95.2%	0.95	36	69.9%	78.5%	0.74
KIR3DL2	78	9	92.0%	95.8%	0.94	31	73.1%	79.0%	0.76	**6**	**92.3%**	**100.0%**	**0.96**
KIR3DL3	80	26	70.1%	94.7%	0.81	31	63.6%	94.2%	0.76	**9**	**88.8%**	**100.0%**	**0.94**
KIR3DP1	74	**17**	82.2%	93.8%	0.88	25	**86.0%**	74.2%	0.80	**17**	78.2%	**100.0%**	**0.88**
KIR3DS1	11	2	84.6%	**100.0%**	0.92	**1**	**100.0%**	90.9%	**0.95**	**1**	**100.0%**	90.9%	**0.95**
All	739	**116**	**88.2%**	**95.4%**	**0.92**	215	82.8%	84.0%	0.83	217	77.6%	92.0%	0.84

Supplementary Table 3: Comparison of Geny, PING and T1K on 40 HPRC samples, using Immuannot [28] annotations. Bold type indicates better results.

Appendix F Comparison With T1K's `t1k-copynumber.py`

The following table presents a preliminary comparison incorporating T1K's unpublished copy number estimation feature (`t1k-copynumber.py`) on 40 HPRC samples against various annotations.

		Geny				T1K				PING			
Gene	Total	Misses	Precision	Recall	F1	Misses	Precision	Recall	F1	Misses	Precision	Recall	F1
KIR2DL1	68	**13**	**85.9%**	93.2%	**0.89**	20	82.9%	87.9%	0.85	46	50.0%	**93.5%**	0.65
KIR2DL2	19	**1**	**94.7%**	**100.0%**	**0.97**	3	85.0%	**100.0%**	0.92	5	93.3%	77.8%	0.85
KIR2DL3	60	**7**	**93.0%**	**94.6%**	**0.94**	15	83.6%	93.3%	0.88	15	90.0%	81.8%	0.86
KIR2DL4	78	**1**	**100.0%**	98.7%	**0.99**	30	68.9%	96.9%	0.81	14	82.1%	**100.0%**	0.90
KIR2DL5A	8	**3**	**72.7%**	**100.0%**	**0.84**	4	66.7%	**100.0%**	0.80	5	66.7%	75.0%	0.71
KIR2DL5B	14	4	**100.0%**	71.4%	0.83	6	66.7%	**100.0%**	0.80	**3**	91.7%	84.6%	**0.88**
KIR2DP1	69	**9**	**93.8%**	92.3%	**0.93**	16	87.1%	89.7%	0.88	35	50.0%	**97.1%**	0.66
KIR2DS1	14	**0**	**100.0%**	**100.0%**	**1.00**	2	87.5%	**100.0%**	0.93	12	**100.0%**	14.3%	0.25
KIR2DS2	17	**1**	**94.1%**	**100.0%**	**0.97**	2	89.5%	**100.0%**	0.94	3	82.4%	**100.0%**	0.90
KIR2DS3	8	1	**100.0%**	87.5%	0.93	1	88.9%	**100.0%**	0.94	**0**	**100.0%**	**100.0%**	**1.00**
KIR2DS4	66	**5**	**95.4%**	96.9%	**0.96**	10	93.8%	90.9%	0.92	9	86.6%	**100.0%**	0.93
KIR2DS5	14	**1**	**100.0%**	92.9%	**0.96**	3	82.4%	**100.0%**	0.90	3	84.6%	91.7%	0.88
KIR3DL1	66	**2**	**97.0%**	**100.0%**	**0.98**	14	85.5%	93.7%	0.89	36	69.9%	78.5%	0.74
KIR3DL2	78	8	**93.3%**	95.9%	0.95	34	64.7%	93.2%	0.76	**7**	91.0%	**100.0%**	**0.95**
KIR3DL3	80	26	70.1%	94.7%	0.81	40	54.8%	95.8%	0.70	**16**	**80.0%**	**100.0%**	**0.89**
KIR3DP1	78	**15**	**86.3%**	92.6%	**0.89**	19	81.0%	94.1%	0.87	16	79.5%	**100.0%**	0.89
KIR3DS1	12	**1**	92.3%	**100.0%**	**0.96**	**1**	92.3%	**100.0%**	**0.96**	2	**100.0%**	83.3%	0.91
All	749	**98**	**90.9%**	**95.3%**	**0.93**	220	77.7%	93.8%	0.85	227	76.3%	91.8%	0.83

Supplementary Table 4: Comparison of Geny, PING and T1K (`t1k-copynumber.py`) on 40 HPRC samples using BAKIR annotations.

		Geny				T1K				PING			
Gene	Total	Misses	Precision	Recall	F1	Misses	Precision	Recall	F1	Misses	Precision	Recall	F1
KIR2DL1	68	**14**	**84.4%**	93.1%	**0.89**	25	75.7%	86.9%	0.81	47	48.8%	**93.3%**	0.64
KIR2DL2	19	**2**	89.5%	**100.0%**	0.94	**2**	90.0%	**100.0%**	**0.95**	4	**100.0%**	78.9%	0.88
KIR2DL3	61	**9**	91.2%	**92.9%**	**0.92**	19	77.6%	**92.9%**	0.85	12	**98.0%**	81.7%	0.89
KIR2DL4	78	**1**	**100.0%**	98.7%	**0.99**	30	68.9%	96.9%	0.81	14	82.1%	**100.0%**	0.90
KIR2DL5A	8	**3**	**72.7%**	**100.0%**	**0.84**	4	66.7%	**100.0%**	0.80	5	66.7%	75.0%	0.71
KIR2DL5B	14	4	**100.0%**	71.4%	0.83	6	66.7%	**100.0%**	0.80	**3**	91.7%	84.6%	**0.88**
KIR2DP1	68	**10**	**90.6%**	93.5%	**0.92**	26	72.9%	87.9%	0.80	36	47.1%	**100.0%**	0.64
KIR2DS1	14	**2**	85.7%	**100.0%**	**0.92**	4	75.0%	**100.0%**	0.86	12	**100.0%**	14.3%	0.25
KIR2DS2	17	**1**	**94.1%**	**100.0%**	**0.97**	4	78.9%	**100.0%**	0.88	3	82.4%	**100.0%**	0.90
KIR2DS3	8	1	**100.0%**	87.5%	0.93	1	88.9%	**100.0%**	0.94	**0**	**100.0%**	**100.0%**	**1.00**
KIR2DS4	67	**4**	**96.9%**	96.9%	**0.97**	9	95.3%	91.0%	0.93	8	88.1%	**100.0%**	0.94
KIR2DS5	14	4	76.9%	90.9%	0.83	7	58.8%	**100.0%**	0.74	**1**	**100.0%**	92.9%	**0.96**
KIR3DL1	66	**2**	**97.0%**	**100.0%**	**0.98**	14	85.5%	93.7%	0.89	36	69.9%	78.5%	0.74
KIR3DL2	79	10	92.0%	94.5%	0.93	35	63.5%	93.1%	0.76	**7**	**92.3%**	**98.6%**	**0.95**
KIR3DL3	80	26	70.1%	94.7%	0.81	35	60.7%	96.2%	0.74	**9**	**88.8%**	**100.0%**	**0.94**
KIR3DP1	78	18	**82.2%**	92.3%	0.87	21	78.5%	93.9%	0.86	**17**	78.2%	**100.0%**	**0.88**
KIR3DS1	12	**1**	92.3%	**100.0%**	**0.96**	**1**	92.3%	**100.0%**	**0.96**	2	**100.0%**	83.3%	0.91
All	751	**112**	**89.1%**	**95.1%**	**0.92**	243	74.8%	93.6%	0.83	216	78.0%	91.8%	0.84

Supplementary Table 5: Comparison of Geny, PING and T1K (`t1k-copynumber.py`) on 40 HPRC samples using SKIRT annotations.

		Geny				T1K				PING			
Gene	Total	Misses	Precision	Recall	F1	Misses	Precision	Recall	F1	Misses	Precision	Recall	F1
KIR2DL1	68	**13**	**85.9%**	93.2%	**0.89**	24	77.1%	87.1%	0.82	46	50.0%	**93.5%**	0.65
KIR2DL2	19	**2**	89.5%	**100.0%**	0.94	**2**	90.0%	**100.0%**	**0.95**	4	**100.0%**	78.9%	0.88
KIR2DL3	60	**8**	91.2%	**94.5%**	**0.93**	20	76.1%	92.7%	0.84	11	**98.0%**	83.1%	0.90
KIR2DL4	74	**5**	**94.8%**	98.6%	**0.97**	34	64.4%	96.7%	0.77	17	78.2%	**100.0%**	0.88
KIR2DL5A	8	**3**	**72.7%**	**100.0%**	**0.84**	4	66.7%	**100.0%**	0.80	5	66.7%	75.0%	0.71
KIR2DL5B	14	4	**100.0%**	71.4%	0.83	6	66.7%	**100.0%**	0.80	**3**	91.7%	84.6%	**0.88**
KIR2DP1	69	**11**	**90.6%**	92.1%	**0.91**	25	74.3%	88.1%	0.81	37	47.1%	**97.0%**	0.63
KIR2DS1	14	**2**	85.7%	**100.0%**	**0.92**	4	75.0%	**100.0%**	0.86	12	**100.0%**	14.3%	0.25
KIR2DS2	17	**1**	**94.1%**	**100.0%**	**0.97**	4	78.9%	**100.0%**	0.88	3	82.4%	**100.0%**	0.90
KIR2DS3	8	1	**100.0%**	87.5%	0.93	1	88.9%	**100.0%**	0.94	**0**	**100.0%**	**100.0%**	**1.00**
KIR2DS4	65	**6**	**93.8%**	96.8%	**0.95**	12	90.6%	90.6%	0.91	9	86.6%	**100.0%**	0.93
KIR2DS5	14	4	76.9%	90.9%	0.83	7	58.8%	**100.0%**	0.74	**1**	**100.0%**	92.9%	**0.96**
KIR3DL1	66	**2**	**97.0%**	**100.0%**	**0.98**	14	85.5%	93.7%	0.89	36	69.9%	78.5%	0.74
KIR3DL2	78	9	92.0%	95.8%	0.94	35	63.5%	93.1%	0.76	**6**	**92.3%**	**100.0%**	**0.96**
KIR3DL3	80	26	70.1%	94.7%	0.81	35	60.7%	96.2%	0.74	**9**	**88.8%**	**100.0%**	**0.94**
KIR3DP1	74	**17**	**82.2%**	93.8%	0.88	21	78.5%	93.9%	0.86	**17**	78.2%	**100.0%**	**0.88**
KIR3DS1	11	2	84.6%	**100.0%**	0.92	2	84.6%	**100.0%**	0.92	**1**	**100.0%**	90.9%	**0.95**
All	739	**116**	**88.2%**	**95.4%**	**0.92**	250	73.9%	93.5%	0.83	217	77.6%	92.0%	0.84

Supplementary Table 6: Comparison of Geny, PING and T1K (`t1k-copynumber.py`) on 40 HPRC samples using Immuannot annotations.
